# Dental, Oral and Craniofacial Tissue Regeneration Consortium (DOCTRC): An infrastructure for accelerating regenerative therapies from discovery to clinical impact

**DOI:** 10.1017/cts.2026.10706

**Published:** 2026-02-13

**Authors:** VyVy Xuan Nguyen, Mutsumi Yoshida, Bridget D. Samuels, William Giannobile, Kevin E. Healy, Michael Jamieson, Nancy Lane, Michael T. Longaker, David J. Mooney, Charles S. Sfeir, Uttam K. Sinha, William R. Wagner, Jeffrey C. Lotz, David H. Kohn, Yang Chai

**Affiliations:** 1 Center for Craniofacial Molecular Biology, Herman Ostrow School of Dentistry, University of Southern California, Los Angeles, CA, USA; 2 Department of Biologic and Materials Sciences & Prosthodontics, School of Dentistry, University of Michigan, Ann Arbor, MI, USA; 3 Harvard School of Dental Medicine, Harvard University, Boston, MA, USA; 4 Department of Bioengineering, University of California Berkeley, Berkeley, CA, USA; 5 University of Southern California, Los Angeles, CA, USA; 6 Center for Musculoskeletal Health, University of California Davis, Davis, CA, USA; 7 Department of Surgery, Division of Plastic and Reconstructive Surgery, Stanford University School of Medicine, Stanford, CA, USA; 8 John A. Paulson School of Engineering and Applied Sciences, Harvard University, Cambridge, MA, USA; 9 Department of Periodontics and Preventive Dentistry and Center for Craniofacial Regeneration, University of Pittsburgh, Pittsburgh, PA, USA; 10 Department of Otolaryngology, USC Keck School of Medicine, Los Angeles, CA, USA; 11 Department of Biomedical Engineering, Swanson School of Engineering, University of Pittsburgh, Pittsburgh, PA, USA; 12 Department of Orthopaedic Surgery, University of California San Francisco, San Francisco, CA, USA

**Keywords:** Translational research, tissue engineering, regenerative medicine, craniofacial research, regulatory science

## Abstract

Translating scientific discoveries in tissue engineering and regenerative medicine (TE/RM) into clinically adopted therapies is hindered by fragmented development pipelines, regulatory and manufacturing challenges, and limited funding. Despite substantial investment by the U.S. National Institutes of Health (NIH), few NIH-funded TE/RM projects achieve commercialization or regulatory approval by the US Food and Drug Administration. The gap between academic innovation and clinical implementation is particularly evident in the dental, oral, and craniofacial (DOC) domain, where market and reimbursement constraints further restrict translation. To address these barriers, the National Institute of Dental and Craniofacial Research established the Dental, Oral and Craniofacial Tissue Regeneration Consortium (DOCTRC), comprising two nationwide Resource Centers tasked with guiding promising technologies from universities and small businesses through preclinical validation toward clinical adoption. This translational science case study outlines DOCTRC’s translational model, highlighting lessons learned from five cohorts of interdisciplinary translational project teams, strategies for navigating manufacturing and regulatory pathways, and approaches for aligning academic innovation with clinical and market needs. The unique impact of the DOCTRC framework demonstrates how disciplined product development activities, non-dilutive funding mechanisms, and a comprehensive support ecosystem can accelerate technology translation, offering a scalable model for other biomedical fields.

## Introduction

The translation of fundamental scientific discoveries into therapies that achieve clinical adoption is impeded by myriad obstacles, including gaps in scientific knowledge, manufacturing difficulties, regulatory constraints, and limited financial resources. An overarching challenge for tissue engineering and regenerative medicine (TE/RM) is that discovery research, product development and clinical practice remain siloed, with insufficient linkages between the technologies developed and unmet clinical needs. While many academic innovations generate *in vivo* efficacy data through traditional funding mechanisms, budgetary constraints become particularly vivid with preclinical validation, including manufacturing scale-up, toxicology studies, and first-in-human (FIH) clinical trials, required for US Food and Drug Administration (FDA) clearance or approval [1]. As a result, it is challenging for university-based RM technologies to reach commercialization and achieve significant clinical impact.

For the past 20 years, the US National Institutes of Health (NIH) have invested ∼$1B annually in RM research [2]. The National Institute of Dental and Craniofacial Research (NIDCR) in particular typically allocates over $40M annually to RM, approximately ∼ 10% of its budget [3]. A portfolio analysis conducted by NIDCR in 2016 revealed that fewer than 5 NIDCR-funded R01 RM projects resulted in FDA submissions, with less than 25% of NIDCR grants pertaining to biomaterials, TE, or RM resulting in patents, a gauge of commercial potential [4]. This outcome is consistent with other areas of medicine, where only 10% of NIH-funded grants generate patents [5].

Central to the NIH mission is the pursuit of fundamental knowledge and its application to improve health. NIH funding has been foundational not only for scientific discoveries but also for advancing them toward clinical application. A review of over 350 new drugs approved by the FDA from 2010 to 2019 found that NIH funding, totaling $187B, contributed to over 99% of these drugs. Notably, more than 80% of the funding supported basic research, with less than 20% directed toward translational activities [6]. Building on this foundation, there is tremendous potential to bring even greater benefits to patients and public health benefits by expanding support for translational and commercialization activities.

While many clinical disciplines face similar constraints, the dental, oral, craniofacial (DOC) space faces unique challenges that further inhibit technology translation, including limited reimbursement, an industry with low risk tolerance, and a smaller pool of investors whose theses/portfolios include DOC technologies. To overcome these barriers, NIDCR established the Dental, Oral and Craniofacial Tissue Regeneration Consortium (DOCTRC) in 2017, comprising two Resource Centers (RCs), the Translational Resource Center (TRC) led by the University of Michigan, University of Pittsburgh, and Wyss Institute at Harvard University and the Center for Dental, Oral and Craniofacial Tissue and Organ Regeneration (C-DOCTOR) led by the University of Southern California, University of California (UC) San Francisco, UC Berkeley, UC Davis, UC Los Angeles, and Stanford University, with the mission to accelerate regenerative therapies for DOC tissues towards commercialization and clinical adoption [7]. At the core of DOCTRC are Interdisciplinary Translational Projects (ITPs), technologies from universities and small companies nationwide, selected based on market potential, anticipated patient value, and probability of clinical adoption.

During each request for proposal cycle, applicants were encouraged to engage with the RCs to expand upon their initial two-page pre-proposals and budget appropriately. The resulting five-page full proposals underwent a rigorous NIH-style review by clinicians and experts in basic science, industry, regulatory affairs, and marketing alongside NIDCR program staff. To further support prospective ITP teams, RCs conducted informational webinars that outlined the essential components of a competitive translational research proposal and highlighted the conceptual distinctions between traditional basic science funding (e.g., NIH R01) and translational product development efforts. Finalists collaborated closely with RC Core Resource Directors and Operations teams to address gaps identified in their proposals, develop project milestones, and refine budgets in advance of presentations to RC leadership, advisors, and NIDCR program staff, which informed ultimate funding decisions. The inaugural cohort of ITPs entered the DOCTRC support ecosystem in 2017, followed by four subsequent groups. Over time, the ITP portfolio was strategically down-selected to concentrate resources on the projects with the highest likelihood of achieving successful FDA submissions within the funding period. Several of the most mature projects have now graduated from the program, giving us considerable insight into the factors that drive team success and enabling early identification and mitigation of common translational pitfalls.

We routinely solicit feedback from ITP teams and members of the broader DOCTRC stakeholder community through surveys and facilitated breakout sessions at our annual meetings. This input directly informs programmatic decision making, guiding the planning of future events, refinement of resources and services, and development of new offerings to address evolving translational research needs. As part of this regular systematic review, sixteen ITPs active at the time of our 5^th^ annual consortium-wide retreat in Bethesda, MD, in October 2024 summarized their progress in three thematic areas: pre-clinical validation and animal model development to address unmet clinical needs, obtaining funding for pre-clinical product development, and interacting with the FDA. After the presentations, breakout sessions were conducted to discuss and identify key lessons learned from the ITPs.

In this translational science case study, we report on the uniqueness, value, and impact of our translational approach, focusing on key lessons learned with case studies, and outline tactics for overcoming challenges in translation. Particularly salient for academic investigators, we share insights in preclinical product development to accelerate technology translation in the DOC TE/RM ecosystem and offer a model extendable to other areas of medicine.

## DOCTRC infrastructure to overcome barriers to translation

Over the past decade, DOCTRC has built a supportive network and infrastructure to guide researchers from academia and small businesses in technology translation, matching them with the full spectrum of resources. Different from the lenses typically used to review basic science projects, translational projects are evaluated and prioritized by DOCTRC according to unmet clinical need, regulatory feasibility, and market potential, each predictors of clinical adoption. Projects with a clear path toward FDA approval are more likely to succeed, and experiments supporting FDA submission are valued over mechanistic studies. Practical concerns, often overlooked in traditional grant reviews, are emphasized.

The DOCTRC program supports preclinical product development through FDA submissions: as described in more detail in the *FDA interactions* section, FDA approval of an Investigational New Drug (IND) or an Investigational Device Exemption (IDE) submission enables the initiation of clinical studies, and clearance of a 510(k) submission allows for the legal marketing of a product. Projects are managed by customized product development teams, employing a milestone-driven phase-gate model [4], where technology development is categorized into five phases (Research & Planning, Feasibility, Verification, Validation, FDA Submission), each with a go/no-go decision based on key deliverables. This structured process spans the entire project lifecycle and integrates aspects of product development including regulatory, intellectual property, commercialization, and manufacturing milestones. ITPs in the DOCTRC program receive comprehensive support, including funding and expert consulting in areas such as large animal models using Good Laboratory Practices (GLP), Good Manufacturing Practices (GMP) scale-up, market analysis, regulatory affairs, quality assurance/quality control, clinical trial design, further funding acquisition, intellectual property protection, and commercialization strategy. Teams are assessed quarterly to provide feedback, address pitfalls, develop alternatives, and implement de-risking strategies to strengthen the likelihood of FDA approvals for first-in-human (FIH) trials. Beyond funding and the valuable feedback provided in quarterly assessments, teams have access to myriad resources and programs available through the RCs, such as the Innovator Networking Seminar Series and training sessions on specific aspects of product and business development. Many of these educational opportunities are publicized and made available to the translational research community at large.

Three unique features of our project management approach are transferable to many other domains. First is the efficiency of our funding model. DOCTRC leverages non-dilutive federal funding with external/industry cost-sharing to de-risk and accelerate commercialization (Section 4). Second is the integration of clinical-regulatory-commercial strategy, which extends beyond translational bootcamps like the National Science Foundation’s i-Corps program, creating a paradigm shift for university-based projects. Regular review by industry, clinical, and scientific advisors ensures that teams prioritize regulatory strategy, commercialization, scale-up, and clinical trial design from the outset. Third is the niche market focus on DOC TE/RM, which offers translatable insights for other fields, addressing challenges unique to non-life-threatening conditions with limited investment appetite.

## Lessons learned in preclinical product development

In our experience, many academic investigators are adept at conducting laboratory phase research and securing R01-type funding, but less so at translating their discoveries to achieve clinical impact. Helping project teams to develop and execute strategic plans for translation while identifying and mitigating risks has therefore been central to DOCTRC’s operation. Tailoring a supportive infrastructure to each ITP’s unique needs and stage of maturation is key to our effectiveness.

For example, as projects were completing small animal proof-of-concept studies, we leveraged existing resources at our DOCTRC participating institutions to develop initial de-risking strategies. As projects neared FDA submissions, we engaged external specialists, most notably regulatory, quality assurance/quality control, and clinical trial consultants, to help ITPs navigate the later stages of preclinical development. Our broad network has enabled teams to engage with advisors with expertise directly relevant to advancing their particular product. Investigators newer to technology translation have especially benefitted from guidance on the design of preclinical animal studies, manufacturing, and clinical trial study design. Described in this context are DOCTRC strategies for accelerating and de-risking innovative translational technologies.

### Preclinical models to address unmet clinical needs

The selection of preclinical models for academic research differs from that needed to adequately address the FDA’s concerns. We highlight lessons learned in animal model development and summarize key points applicable to other translational investigations: model selection and study design incorporating clinically relevant outcome measures, and considerations when interacting with the FDA.

A first case study involves an ITP developing bioinspired scaffolds for craniofacial muscle loss with functional impairment. Original work demonstrated nearly complete structural and functional recovery using a tibialis anterior model. The shift to regeneration of a craniofacial muscle was met with challenges of vastly different scale (masseter vs. tibialis anterior) and muscle regeneration kinetics. A new craniofacial model was needed to address the site-specificity in healing rate. An anatomically relevant model was established in a rat [8], and the team subsequently developed a sheep protocol. Rigorous cell- and tissue-based metrics for evaluating muscle repair, regeneration, and functional recovery were established and optimized specifically for the masseter. Key lessons learned include that dynamics, such as regeneration kinetics, may not generalize across different anatomies, and that the development of musculoskeletal therapeutics may require careful consideration of the unique properties of the pre-clinical injury model.

In another case, an ITP with technology for endodontic therapy came onboard following *in vitro* validation that their engineered bioinspired hydrogel regenerated dental pulp constructs similar to native, healthy pulp tissue [9–11] to launch a study in a canine model. While few clinicians have experience conducting endodontic treatments on canines, the consortium connected the team with an experienced clinician. Positive data warranted further pursuit, but terminal studies using companion animals have been prohibited in many states. Consortium regulatory experts advised requesting FDA approval to advance directly to a small FIH study with hydrogel manufactured under current GMPs. This FDA submission is scheduled in 2026, with ongoing fundraising to initiate the clinical study in late 2026. Alternatives to (companion) animals should be considered and can lead to a quicker path to clinical studies. With the current US regulatory climate, this strategy may become more common.

### Funding preclinical product development

**Figure 1.**
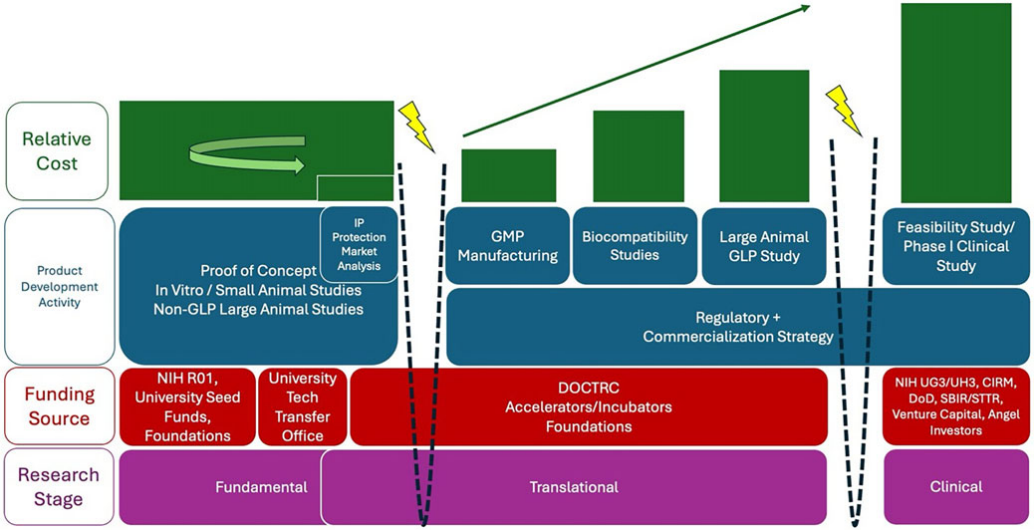
Relative costs of product development activities. The fundamental research stage can be lengthy, iterative, costly, and risky ending at the first “valley of death” (represented by the dotted lines and lightning bolts). DOCTRC bridges this gap by supporting the transition to translational preclinical product development, beginning with market analysis and initial regulatory strategy, followed by successive stages of preclinical activities punctuated by quarterly 360-degree review and go/no-go gates. Proof-of-concept studies can be leveraged to obtain funding from external accelerators/incubators and foundations to supplement DOCTRC support of translational activities. The costs and pace of these translational product development activities ramp up considerably relative to fundamental proof of concept studies. A second “valley of death” exists once a product receives FDA approval for in-human use but has no funding to execute clinical trials.

Finances are a considerable obstacle in the development of regenerative therapies. In the US, the development of a medical device can cost $54 million [12] and that of a new drug can exceed ∼$2.6 billion [13], with costs rising from proof-of-principle through GLP large animal studies and GMP scale-up (Figure 1). With this in mind, we aim to help ITPs maximize funding and demonstrate product value to potential investors by encouraging: (1) focusing on one strategic initial indication, (2) streamlining regulatory and commercialization strategies, and when appropriate (3) phased development in pursuit of broader indications. Together, these guidelines help maintain budgetary focus and avoid “mission creep” that distracts from the goal of FDA approval.

Here we highlight two ITPs where refinement of the clinical indication proved efficient: (1) verteporfin for scar prevention during cleft lip scar revision; and (2) a resorbable hydrogel for pulp revitalization in immature permanent teeth. Both products have broad potential applications (e.g., hypertrophic scarring/wound healing and adult populations, respectively). While pursuing additional indications or larger markets may be appealing scientifically and commercially, focusing on a single strategic indication allows the project teams to concentrate time and resources toward regulatory clearance and market entry, which can later be leveraged for scope expansion. Available FDA opportunities, such as ‘fast-track’ or accelerated approval mechanisms and orphan drug designation, may figure into determination of the initial indication to pursue.

The advantages of streamlined regulatory and commercialization pathways are exemplified by two projects that originally utilized stem cells for their proof-of-concept studies. The projects both aimed to regenerate bone: calvarial bone using stem cells loaded onto a 3D-printed scaffold, and alveolar bone lost to peri-implantitis using stem cells in an injectable hydrogel. Both projects avoided biologic-device combination product classification by switching from using stem cells to autologous bone marrow aspirate, significantly reducing regulatory complexity and costs. Another successful approach is drug repurposing using the 505(b)2 pathway, through which some teams shortened their path to FDA approval after obtaining the drug master file from the original sponsor. When access to the master file is not available, experts can advise whether developing a new formulation, potentially yielding a favorable intellectual property position, may be prudent.

A focused regulatory strategy can also enable future product iterations and fundraising. In the case of the 3D-printed scaffold for calvarial bone regeneration, the team aims to receive IDE approval for a standard-size scaffold, which could then be used in future 510(*k*) applications as a predicate device for defect-specific scaffolds. Similarly, another ITP is pursuing a “stepwise” regulatory approach of Class I registration of their initial product, followed by a 510(k) for another product variation. These examples illustrate savvy regulatory strategy and financial prudence are often complementary.

As DOCTRC does not support clinical studies and ITP budgets alone may not cover full preclinical development, securing other sources of funding is critical. Customer discovery and feedback from key opinion leaders further de-risk technologies and strengthen attractiveness to investors. A team developing a neuromimetic hydrogel for salivary gland regeneration secured support from the California Institute for Regenerative Medicine to complete GLP studies and manufacturing scale-up to enable an IND submission. Another team developing ultrasound-assisted non-viral gene therapy for radiation-induced xerostomia obtained funding from a biomedical innovation fund at their home institution to support their GLP studies. Several other teams, including those developing a distraction osteogenesis device, hydrogel for endodontic regeneration, regenerative adhesive for 3-wall tooth socket reconstruction, and particles for tooth remineralization, obtained NIH and/or National Science Foundation Small Business Innovation Research (SBIR) and Small Business Technology Transfer (STTR) funding. Data and strategies developed with DOCTRC support underpinned these awards. Similarly, continued guidance and funding of IND-enabling studies helped the team developing a focal adhesion kinase inhibitor hydrogel to generate data for a successful Department of Defense grant in support of the FIH trial of their product to prevent scar formation. Given that many prospective industry partners in the DOC space are seeking technologies with promising FIH data already in hand, raising funds to obtain such data is critical for eventual commercialization and clinical adoption. Even when research and product development are accelerated to achieve FDA clearance or approval, a major funding gap remains before a new product can make a clinical impact.

### FDA interactions

For products deemed to be of higher risk, an IND or IDE package with extensive documentation is required, including: (1) preclinical data demonstrating reasonable safety in humans, (2) information ascertaining consistent manufacturing, and (3) FIH testing plans. For devices with established predicates for the same intended use and similar technological/material composition/performance, a more expedient, less onerous 510(k) pathway is available. Although the 510(k) pathway does not require premarket clinical trials, companies may seek such data to drive adoption.

For novel technologies, precedents of established animal models and clinical endpoints often do not exist, and an appropriate regulatory pathway is not always obvious. We encourage early engagement of ITPs with the FDA, mediated by a seasoned regulatory professional. Mechanisms such as (pre-) Request for Designation (RFD) and pre-submission, pre-IND, and Initial Targeted Engagement for Regulatory Advice on CBER/CDER ProducTs (INTERACT) meetings (for devices, drugs, and biologics, respectively) are central to preparing strong IND/IDE/510(k) submissions. A comprehensive understanding of FDA requirements specific to the product in development is critical to minimize complications that can increase costs and cause delays. For example, one ITP proposed a single-species GLP toxicology study, whereas the FDA requested a second species. In another case, a team developed a regulatory strategy based on a pre-submission meeting where additional preclinical data was not required, but upon 510(k) submission, a new concern was raised, necessitating development plan revision. While unexpected outcomes may occur, early course corrections and planning can help mitigate setbacks.

In certain cases, securing a less burdensome pathway with the support of an experienced regulatory professional can prevent costly missteps and jeopardized commercialization opportunities. Without established precedents, teams developing novel products must collaborate with the FDA to define an acceptable IND/IDE package. One project team iterated with the FDA to converge on the appropriate animal model. In response to data using a juvenile porcine model (due to the cost and logistical challenges of housing/imaging fully grown swine) presented at a pre-submission meeting, the FDA requested data from aged animals to better reflect the age range of the proposed clinical trial subjects. The team convincingly argued that an aged rabbit model would suffice to demonstrate safety and efficacy by interpolating results from pilot studies and publications that had used aged rabbits, avoiding increased study cost and complexity using a larger farm animal.

Through strategic engagement with the FDA led by experts, regulatory risk can be reduced, while operating on a limited budget. This deliberate effort has allowed us to maximize return on investment, achieving results in a faster and more cost-efficient manner than similar accelerator/incubator programs, as discussed in the following section.

## RC/ITP accomplishments

Since 2017, the two RCs have reviewed over 200 pre-proposals, invited full proposal submission from approximately half, and ultimately funded 44 projects. To date, about two-thirds have exited the program, either upon meeting graduation criteria (licensing, substantial follow-on funding, FDA clearance/approval, or commercialization) or not meeting milestones. Exited projects remain part of the consortium and continue to benefit from guidance and participation in various training activities. Quarterly project assessments provide a structured opportunity for refinement of our evaluation framework, enabling systematic identification of common factors contributing to project exit and/or failure to achieve predefined milestones. In many cases, missed milestones reflected an inability to meet established “go/no-go” criteria for specific pre-clinical validation metrics. Insights gained through this iterative process inform adjustments to the feedback, guidance, and recommendations provided to active projects.

Importantly, ITP feedback consistently reveals that even exited projects that fell short on their milestones nonetheless benefited from participation in the program, gaining valuable experience and advice, and several have advanced their technologies with other funding. For example, a project on engineered lips secured Department of Defense funding for continued preclinical advancement.

**Figure 2.**
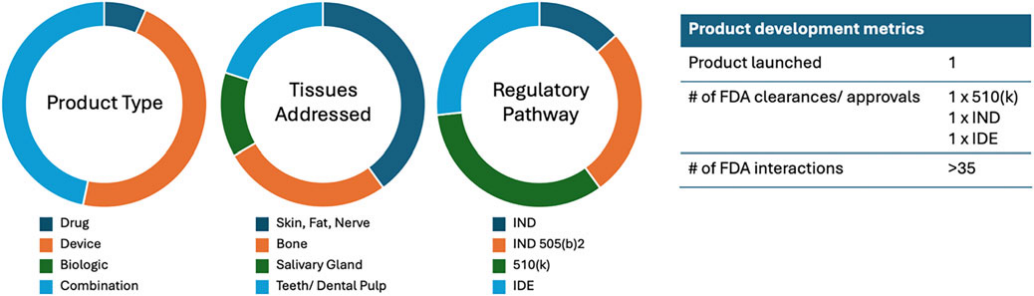
Current DOCTRC project portfolio. Current portfolio comprises 15 projects from academic institutions and small companies across the U.S. Tissues addressed by portfolio technologies span across the DOC complex, with varied product type and regulatory pathways (left). Key product development metrics of current projects are included in the table (right).

Our current portfolio includes 15 projects, addressing unmet needs across a variety of DOC indications (Figure 2). Many of these projects can be extended to other tissues, including musculoskeletal, nervous, and gastrointestinal systems, and address broader challenges like inflammation, infection, wound healing, trauma, and congenital malformations. As oral health is intimately linked to overall health, viewing these projects in that context underscores the generalizability of our approach.

With DOCTRC support, 15 project teams have conducted a total of 35 formal interactions with the FDA to date, including RFD and pre-submission/INTERACT meetings. Of note, one product is already on the market, and additional 510(*k*) clearance and IND/IDE submissions are in progress, with one project team scheduled to initiate a clinical study designed with DOCTRC support in early 2026. Many of the other projects are on target to submit 510(k) or IND/IDE packages in the first half of 2026.

**Figure 3.**
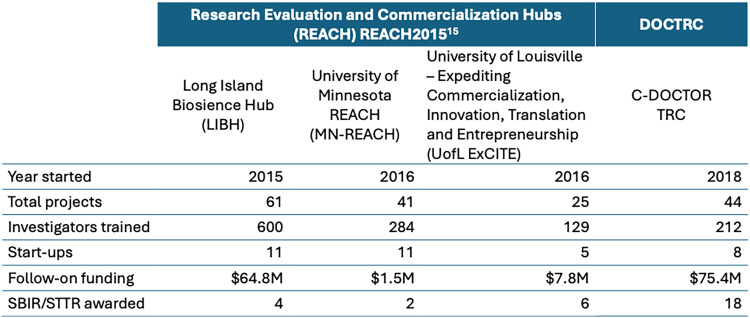
Comparison of DOCTRC program metrics to previous NIH-supported translational programs. Key translational metrics for DOCTRC program are comparable to or exceed those of similar programs, such as the research evaluation and commercialization hubs (REACH) [15]. In particular, follow-on funding and number of SBIR awarded to projects funded by DOCTRC are notable.

DOCTRC ITPs have collectively received 65 follow-on grants/awards/investments totaling $75.4M in leveraged external funding, representing a 2.5-fold financial return on investment by DOCTRC (Figure 3). A total of 18 SBIR/STTR grants have been awarded to ITPs with a remarkable 80% success rate, compared to a national average of ∼ 13% [14]. Beyond SBIR/STTR funding, most investments have not come from NIH, signifying that the DOCTRC approach positions technologies to be highly competitive for third-party funding and facilitates efficient transition from federal to non-federal support. Considering a typical annual NIH R01 grant budget of $250,000, one team brought a product to market and attained 510(k) clearance with an investment equivalent to 2–3 years of R01-level funding. Another project achieved an IND approval with funding equivalent to a 5-year R01 grant. The DOCTRC program continues to meet its goal of overcoming the translational limitations of the R01 funding mechanism with great efficiency, even compared to other federal programs targeting biomedical translation, such as the NIH Research Evaluation and Commercialization Hubs (REACH) [15] (Figure 3). Collectively, these programs support the translation of discoveries into products to benefit public health and provide the further benefit of training a workforce in technology commercialization and entrepreneurship, supporting economic development.

## Conclusion

The gap between academic innovation and successful therapies in TE/RM highlights the dire need for translational infrastructure that supports the full pathway from discovery to commercialization and clinical adoption. DOCTRC addresses this need through a comprehensive, milestone-driven model that provides teams with financial support and connections to resources vital to preclinical product development. Integration of these customized resources from the start has enabled efficient technology de-risking to enhance the likelihood of securing follow-on investment, regulatory approval, and ultimately widespread clinical adoption.

Key lessons learned (Table 1) in DOCTRC preclinical product development underscore the importance of:

1. Strategically selecting preclinical models to address FDA perspectives and demonstrate clinically relevant outcomes.
2. Developing customized strategies for each project, focusing on a minimum viable product to manage costs and timeline.
3. Early and effective engagement with the FDA to align on development plans and refine strategies, thereby avoiding costly roadblocks.

**Table 1.** Lessons learned in preclinical product development. Summary of key lessons learned and approaches taken by DOCTRC and supported teams in response to barriers in three areas of preclinical product development: (1) selection and design of preclinical models, (2) funding preclinical product development, and (3) FDA interactions

	Barriers	Lessons learned/approaches
Pre-clinical models to address unmet clinical needs	Selection of preclinical models for academic research differs from that needed to address FDA safety concerns – Assays and endpoints in basic science animal research may differ from relevant clinical outcomes – Use of previously common model organisms (such as companion animals) to demonstrate safety and efficacy are prohibited	Map unique anatomical and functional assays to relevant clinical outcomes in FDA-recognized pre-clinical injury models Engage with the FDA to explore New Approach Methods or feasibility of entering into first-in-human studies with reduced animal testing may be useful as the US regulatory climate seeks to reduce animal testing in research. Emerging lab-on-a-chip models may be considered as surrogates or substitutes for animal testing.
Funding preclinical product development	Funding for preclinical product development and clinical testing is often limited, and it is critical for investigators to maintain budgetary focus and manage scientific scope to achieve regulatory clearance as soon as possible – Pursuit of broad applications, while useful, may result in complicated regulatory pathways and subsequent delays related to inefficient use of time and resources The costs and pace of translational product development activities increase considerably over time – Often one source of funding is not sufficient to cover all aspects of product development	Focus on one strategic initial indication despite potential for broad applications to ensure efficient allocation of time and resources toward regulatory clearance Streamlining of regulatory and commercialization strategies to avoid more complicated drug- or biologic-device combination product regulatory pathways; pursue agreement with original sponsor for access to drug master file to shorten regulatory pathway in the case of repurposed drugs Consider phased product development which can allow for the clearance of an initial product which can then serve as a predicate device in future 510(k) submissions Build a strong proof-of-concept and engage in continual de-risking to position project to obtain additional funding for further pre-clinical development activities clinical studies
FDA interactions	IND/IDE/510(k) submissions are extensive and often novel to academic investigators – Developing clear regulatory strategies with experienced regulatory professionals in conjunction with the FDA can mitigate setbacks such as increased costs, additional required studies, and missed opportunities	Engage with the FDA through mechanisms like pre-RFD, INTERACT, pre-IND, and pre-submission meetings, which serve as opportunities to course-correct and even negotiate FDA interactions offer opportunities to collaboratively define and align on an acceptable IND/IDE/510(k) package

While DOCTRC specializes in the DOC TE/RM space, the model holds promise in broader areas of medicine where similar translational bottlenecks exist. With a non-dilutive funding model that leverages cost-sharing and an integrated strategy to advance novel technologies, the program has supported investigators’ transition from discovery-stage research to technology translation towards commercialization and clinical adoption. In doing so, DOCTRC maximizes the return on public research investments by accelerating improvements in patient care through regenerative therapies.
